# Lysosome repositioning as an autophagy escape mechanism by *Mycobacterium tuberculosis* Beijing strain

**DOI:** 10.1038/s41598-021-83835-4

**Published:** 2021-02-22

**Authors:** Thanida Laopanupong, Pinidphon Prombutara, Phongthon Kanjanasirirat, Salisa Benjaskulluecha, Atsadang Boonmee, Tanapat Palaga, Stephane Méresse, Jiraporn Paha, Tegar Adriansyah Putra Siregar, Tanawadee Khumpanied, Suparerk Borwornpinyo, Angkana Chaiprasert, Pongsak Utaisincharoen, Marisa Ponpuak

**Affiliations:** 1 Department of Microbiology, Faculty of Science, Mahidol University, Rama VI Road, Bangkok, 10400 Thailand; 2 Omics Sciences and Bioinformatics Center, Faculty of Science, Chulalongkorn University, Bangkok, Thailand; 3 Microbiome Research Unit for Probiotics in Food and Cosmetics, Faculty of Science, Chulalongkorn University, Bangkok, Thailand; 4 Excellent Center for Drug Discovery, Faculty of Science, Mahidol University, Bangkok, Thailand; 5 Inter-Disciplinary Graduate Program in Medical Microbiology, Graduate School, Chulalongkorn University, Bangkok, Thailand; 6 Department of Microbiology, Faculty of Science, Chulalongkorn University, Pathumwan, Bangkok, Thailand; 7 Aix Marseille University, CNRS, INSERM, CIML, Marseille, France; 8 Department of Biotechnology, Faculty of Science, Mahidol University, Bangkok, Thailand; 9 Drug-Resistance Tuberculosis Research Fund, Siriraj Foundation, Faculty of Medicine Siriraj Hospital, Mahidol University, Bangkok, Thailand; 10 Office of Research and Development, Faculty of Medicine Siriraj Hospital, Mahidol University, Bangkok, Thailand; 11 Pornchai Matangkasombut Center for Microbial Genomics, Department of Microbiology, Faculty of Science, Mahidol University, Bangkok, Thailand

**Keywords:** Immunology, Microbiology

## Abstract

Induction of host cell autophagy by starvation was shown to enhance lysosomal delivery to mycobacterial phagosomes, resulting in the restriction of *Mycobacterium tuberculosis* reference strain H37Rv. Our previous study showed that strains belonging to *M. tuberculosis* Beijing genotype resisted starvation-induced autophagic elimination but the factors involved remained unclear. Here, we conducted RNA-Seq of macrophages infected with the autophagy-resistant Beijing strain (BJN) compared to macrophages infected with H37Rv upon autophagy induction by starvation. Results identified several genes uniquely upregulated in BJN-infected macrophages but not in H37Rv-infected cells, including those encoding Kxd1 and Plekhm2, which function in lysosome positioning towards the cell periphery. Unlike H37Rv, BJN suppressed enhanced lysosome positioning towards the perinuclear region and lysosomal delivery to its phagosome upon autophagy induction by starvation, while depletion of Kxd1 and Plekhm2 reverted such effects, resulting in restriction of BJN intracellular survival upon autophagy induction by starvation. Taken together, these data indicated that Kxd1 and Plekhm2 are important for the BJN strain to suppress lysosome positioning towards the perinuclear region and lysosomal delivery into its phagosome during autophagy induction by starvation to evade starvation-induced autophagic restriction.

## Introduction

Tuberculosis is ranked number one among infectious diseases with an estimated 10 million new cases and 1.5 million deaths annually worldwide^1^. One third of the global population is latently infected with *Mycobacterium tuberculosis* and more than 95% of tuberculosis-associated deaths occur in low- and middle-income countries^2^. The only available tuberculosis vaccine, dubbed BCG, can prevent severe tuberculosis in children but it does not protect against pulmonary tuberculosis in adults, while the emergence and rapid spread of multidrug-resistant and extensively drug-resistant *M. tuberculosis* strains have posed a serious threat to the global tuberculosis control program^2^. Difficulties in rapid diagnosis and inadequacy of drugs to treat drug-resistant tuberculosis are the main barriers for effective control^3^. Therefore, new drugs are urgently needed.

Global distribution and emergence of drug-resistant tuberculosis are associated with *M. tuberculosis* genotypic variation^4–8^. The Beijing family, notorious for drug-resistance and hyper-virulence, now represents around 50% of the strains in East Asia and more than 13% of strains worldwide^5^, with an increasingly wide spread of the Beijing genotype^4,9^. The reasons for its high transmissibility are unclear. Previous studies showed that the Beijing genotype had greater ability to survive inside host macrophages, causing high bacterial load and greater mortality rates in animal models, with high acid-fast bacilli (AFB) smear-positive sputum in human patients^6,10–14^. However, the molecular mechanisms and factors underlying this increased ability of the Beijing genotype to survive in the host remain to be determined. Recently, we reported that Beijing strains had a previously unrecognised ability to evade elimination by host autophagy, an important innate immune mechanism against *M. tuberculosis* in host macrophages^15^.

Autophagy is a conserved lysosomal-dependent degradation process that has been demonstrated to play a key role in defending against intracellular bacteria, viruses and protozoan parasites^16–19^. Autophagy of microbes is initiated by a stress signal such as starvation of the infected cells due to consumption of host cell amino acids by the pathogens and presence of pathogen-associated molecular patterns and host cytokines^17,20,21^. During autophagy, double-membrane autophagosomes engulf cytosolic substrates such as whole pathogens or their components and deliver them along microtubules to fuse with acidic lysosomes concentrating at the perinuclear region, resulting in the delivery of lysosomal hydrolases to digest the enclosed contents^22^.

In the context of *M. tuberculosis* infection, induction of autophagy by starvation or other autophagy inducers resulted in the death of intracellular *M. tuberculosis* reference strains such as H37Rv and strains belonging to the East African Indian genotype^15,23–29^, even though they have blocked the phagolysosome biogenesis and other host cell defence mechanisms^30^. Our previous study showed that unlike H37Rv and East African Indian strains, which were eliminated by starvation-induced autophagic restriction in host macrophages dependent upon Beclin-1, the Beijing strains were able to resist starvation-induced autophagic control by host cells^15^. In the same study, we also revealed that evasion of the Beijing strains from starvation-induced autophagic restriction was not simply achieved by blocking the autophagy-mediated acidification of their phagosomes or by inhibiting the general autophagic flux in host cells but by blocking lysosomal delivery into their phagosomes upon autophagy induction by starvation^15^. We found that while lysosomes were delivered to the phagosomes of H37Rv and East African Indian strains upon autophagy induction by starvation dependent upon Beclin-1, lysosomal delivery to phagosomes of autophagy-resistant Beijing strains was inhibited^15^. However, the factors involved were unknown.

In this study, we attempted to determine the host factors contributing to escape of the autophagy-resistant *M. tuberculosis* Beijing strain (BJN) from starvation-induced autophagic restriction by conducting RNA-Seq analyses of host macrophages infected with BJN compared to those infected with H37Rv upon autophagy induction by starvation. Our results identified several genes that were differentially regulated in BJN-infected macrophages during autophagy induction by starvation but not in H37Rv-infected cells. The findings were confirmed by qRT-PCR. In silico gene ontology (GO) analysis showed that several pathways were enriched in BJN-infected macrophages upon autophagy induction by starvation, including the lysosome localisation pathway. Two of the genes in this pathway, *Kxd1* and *Plekhm2*, were previously shown to function in lysosome positioning towards the cell periphery^31^; therefore, we tested whether the BJN strain induced lysosome relocation in host macrophages during autophagy induction by starvation. High-content image analysis showed that in contrast to H37Rv, the BJN strain suppressed enhanced lysosomal positioning towards the perinuclear region and lysosomal delivery to mycobacterial phagosomes upon autophagy induction by starvation. Depletion of Kxd1 and Plekhm2 expressions reverted the aforementioned effects and BJN intracellular survival reduced upon autophagy induction by starvation. Our study identified a new strategy for evasion of autophagic restriction by *M. tuberculosis* that potentially could be targets for the discovery of new drugs against this emerging disease.

## Results

### Gene expression quantification

**Figure 1:**
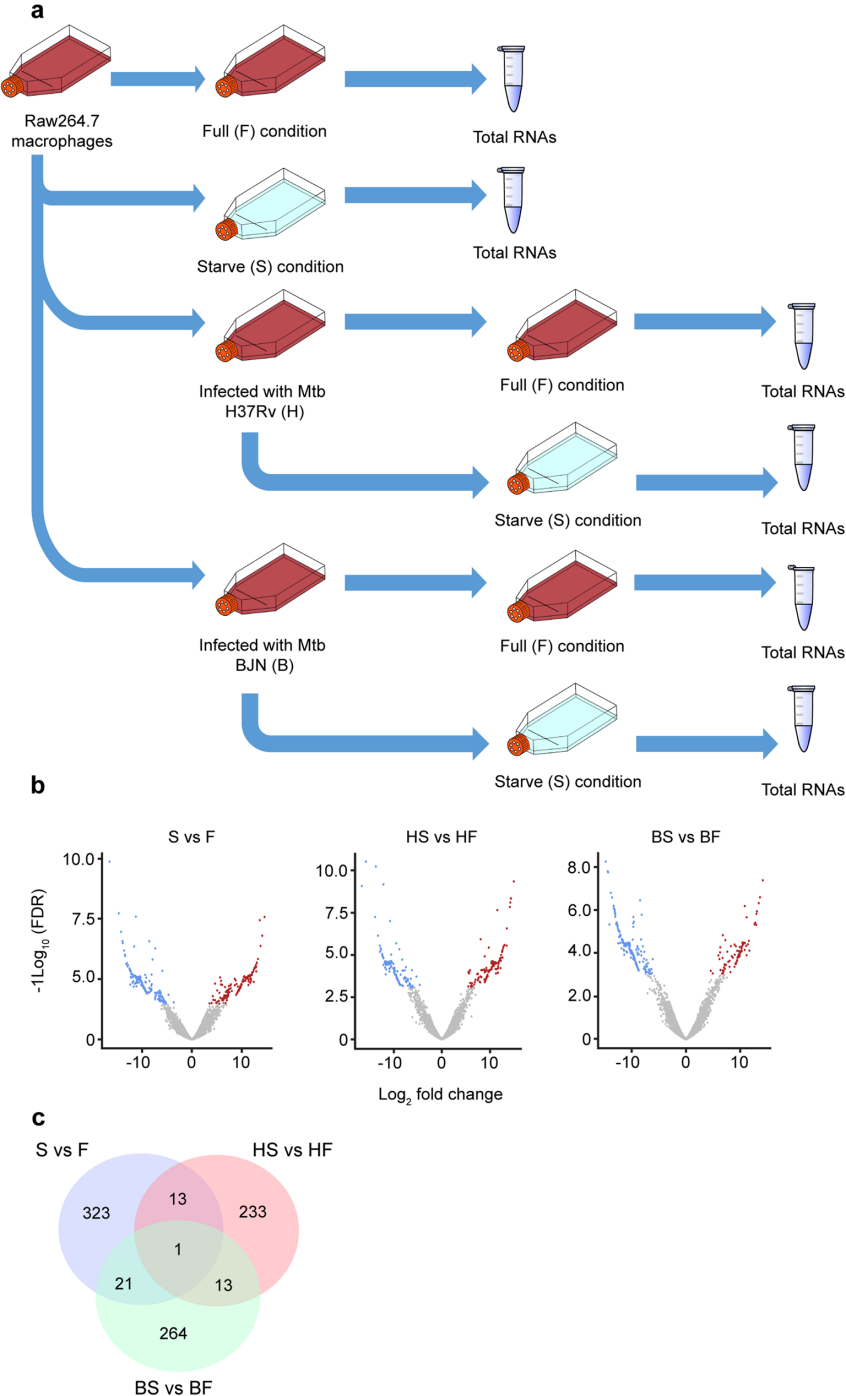
Differential transcript expression analysis. (**a**) Raw264.7 macrophages were infected with or without the autophagy-sensitive *M. tuberculosis* reference strain H37Rv or the autophagy-resistant Beijing strain (BJN) followed by autophagy induction by starvation. Differentially expressed transcripts were then determined among the three biological conditions (experimental group versus baseline; S vs F, HS vs HF and BS vs BF). (**b**) Volcano plots were used to determine significantly different expressed transcripts. Only log_2_ (fold change) ≥ 2 or ≤ 2 and false discovery rate (FDR) adjusted p-values < 0.05 were analysed. Downregulated transcripts were labelled in blue, while upregulated transcripts were labelled in red. (**c**) A Venn diagram was used to illustrate the numbers of differentially expressed transcripts that were common or unique among different conditions. *F* macrophages without infection, *S* macrophages without infection followed by autophagy induction by starvation, *HF* macrophages infected with H37Rv without autophagy induction, *HS* macrophages infected with H37Rv followed by autophagy induction by starvation, *BF* macrophages infected with BJN without autophagy induction, *BS* macrophages infected with BJN followed by autophagy induction by starvation.

Our previous study showed that *M. tuberculosis* Beijing strains were able to resist starvation-induced autophagic elimination in host macrophages but the factors and mechanisms involved were unclear^15^. We employed RNA-Seq technology to profile global gene expression levels in RAW264.7 macrophages infected with different mycobacteria induced to undergo autophagy by starvation. RNA-Seq analyses were performed under three biological conditions: (1) macrophages induced to undergo autophagy by starvation without infection (S versus F), (2) macrophages infected with the autophagy-sensitive H37Rv strain followed by autophagy induction by starvation (HS versus HF) and (3) macrophages infected with the autophagy-resistant Beijing strain (BJN) followed by autophagy induction by starvation (BS versus BF). Each condition had two independent biological replicates (12 samples in total) (Fig. 1a). Approximately, 420 million raw sequencing paired-end reads were generated and subjected to adaptor trimming and low quality read filtering (quality score < 20). Subsequently, an average of 33.4 million clean reads per sample was mapped to the mouse reference genome, GRCm38 (GCA_000001635.8). Concordant pair alignment ranged between 76.7% and 86.6% (Supplementary Table S1 and Supplementary Dataset S1).

To identify possible resistance factors to starvation-induced autophagic restriction by the BJN strain, genome-wide expression changes between each condition were compared. Significantly different expression results (FPKM > 10 per sample and a false discovery rate (FDR) < 0.05) for the three biological conditions are displayed as volcano plots in Fig. 1b and summarised in Table 1. A detailed list of differentially expressed genes is also presented as Supplementary Dataset S2. From this list, seven genes were selected to validate our RNA-Seq results by qRT-PCR, with results concurring with our RNA-Seq analyses (Supplementary Fig. S1). To identify differentially expressed genes (DEGs) that were uniquely altered only in macrophages infected with the BJN strain subjected to autophagy induction by starvation, we conducted a Venn diagram analysis. Results showed that 264 DEGs were uniquely altered only in the BS versus BF condition (Fig. 1c).

**Table 1:** Summary of the differentially expressed transcripts between conditions.

Condition	S vs F	HS vs HF	BS vs BF
Up-regulated	155	125	86
Down-regulated	203	135	213
Total	358	260	299

### Function and pathway enrichment analyses

To obtain a better insight into the roles of these uniquely altered 264 DEGs, we carried out function and pathway enrichment analyses based on the Kyoto Encyclopaedia of Genes and Genomes (KEGG)^32–34^ and gene ontology (GO) databases using the DAVID Bioinformatics Resource 6.8. Enrichment results are shown in Table 2. Significant (p-value < 0.05) GO terms with more than tenfold enrichment included the protein initiator methionine removal (GO:0070084), regulation of germinal centre formation (GO:0002634), nuclear-transcribed mRNA catabolic process (GO:0000956), lysosome localisation (GO:0032418), entrainment of circadian clock by photoperiod (GO:0043153), protein-L-isoaspartate (D-aspartate) O-methyltransferase activity (GO:0004719) and Y-form DNA binding (GO:0000403). Our previous data showed that autophagy-resistant Beijing strains suppressed lysosomal delivery into their phagosomes during autophagy induction by starvation^15^; therefore, the lysosome localisation pathway (GO:0032418) attracted our interest. The genes listed in this pathway included *KxDL motif containing 1* (*Kxd1*), *Pleckstrin homology domain containing family M* (*with RUN domain*) *member 2* (*Plekhm2* or *Skip*) and *SNAP-associated protein* (*Snapin*).

**Table 2:** GO analyses of the uniquely altered BS vs BF transcripts.

GO ID	GO term	Gene name	Fold enrichment	p-value
**Biological pathway**				
GO:0070084	Protein initiator methionine removal	*Metap2, Metap1d*	56.33021807	0.03492627
GO:0002634	Regulation of germinal centre formation	*Mef2c, Rc3h1*	42.24766355	0.04629641
GO:0000956	Nuclear-transcribed mRNA catabolic process	*Ptbp1, Rc3h1, Mrto4*	18.10614152	0.01144972
GO:0032418	Lysosome localisation	*Plekhm2, Kxd1, Snapin*	15.84287383	0.01486726
GO:0043153	Entrainment of circadian clock by photoperiod	*Rbm4b, Pml, Ppp1cc*	12.07076101	0.02503616
GO:0006611	Protein export from nucleus	*Xpo4, Eif5a, Stradb*	8.740895907	0.04553277
GO:0016575	Histone deacetylation	*Morf4l2, Hdac9, Suds3*	8.740895907	0.04553277
GO:0043124	Negative regulation of I-κB kinase/NF-κB signaling	*Ash1l, Casp8, Ppm1b, Tnip1*	8.666187395	0.01078362
GO:0009409	Response to cold	*Rbm3, Casp8, Zfp516, Hspd1*	8.44953271	0.01155847
GO:0009411	Response to UV	*Men1, Ercc8, Primpol, Pml*	7.191091668	0.01786757
GO:0032922	Circadian regulation of gene expression	*Rbm4b, Pml, Ppp1cc, Mybbp1a*	5.540677187	0.03521088
GO:0006417	Regulation of translation	*Rbm4b, Tnrc6c, Cpeb2, Klhl25, Rbm3, Pum2, Mif4gd*	4.888159419	0.00311389
GO:0016569	Covalent chromatin modification	*Ehmt1, Brd3, Morf4l2, Phf20, Nr3c1, Cbx6, Men1, Mbtd1, Mtf2, Jmjd6, Ash1l, Kdm4a, Hdac9, Suds3*	4.447122479	1.70E-05
GO:0000209	Protein polyubiquitination	*Ercc8, Syvn1, Tnks, Ube3c, Rnf19b*	4.101714908	0.03355292
**Cellular component**				
GO:1904115	Axon cytoplasm	*Snapin, Rangap1, Dst*	8.416952055	0.04882424
GO:0005643	Nuclear pore	*Xpo4, Eif5a, Tnks, Rangap1*	5.61130137	0.03416832
GO:0016363	Nuclear matrix	*Men1, Ercc8, Ppig, Pml, Srpk1*	4.627877418	0.0228822
GO:0000784	Nuclear chromosome, telomeric region	*Men1, Pml, Orc4, Tnks, Wrn, Ppp1cc*	4.241613634	0.01367434
GO:0005815	Microtubule organizing centre	*Cdc42, Casp8, Tmub1, Birc6, Pde4dip, Nr3c1, Nek7*	4.054617764	0.00772725
GO:0055037	Recycling endosome	*Nisch, Vipas39, Tbc1d14, Tmub1, Atp11a*	3.869863014	0.04029551
GO:0005769	Early endosome	*Nisch, Ankrd13b, Vipas39, P2ry2, Snx1, Atp11a, Ptpn1, Hspd1*	3.178082192	0.01325987
**Molecular function**				
GO:0004719	Protein-l-isoaspartate (d-aspartate) O-methyltransferase activity	*Pcmtd2, Pcmt1*	55.91666667	0.03517687
GO:0000403	Y-form DNA binding	*Men1, Wrn*	41.9375	0.04662664
GO:0030145	Manganese ion binding	*Cdipt, Galnt1, Wrn, Ppm1b*	7.138297872	0.01820486
GO:0003714	Transcription corepressor activity	*Rcor3, Hipk3, Hipk2, Hdac9, Hsbp1, Mybbp1a*	3.205414013	0.03938472
**KEGG pathway**				
mmu00270	Cysteine and methionine metabolism	*Got1, Ahcyl2, Amd1, Mdh2*	9.155952381	0.00898345
mmu04120	Ubiquitin mediated proteolysis	*Ercc8, Syvn1, Xiap, Pml, Birc6, Ube3c*	3.868712274	0.01837515

The function and pathway enrichment analyses were conducted using DAVID Bioinformatics Resource 6.8.

**Figure 2:**
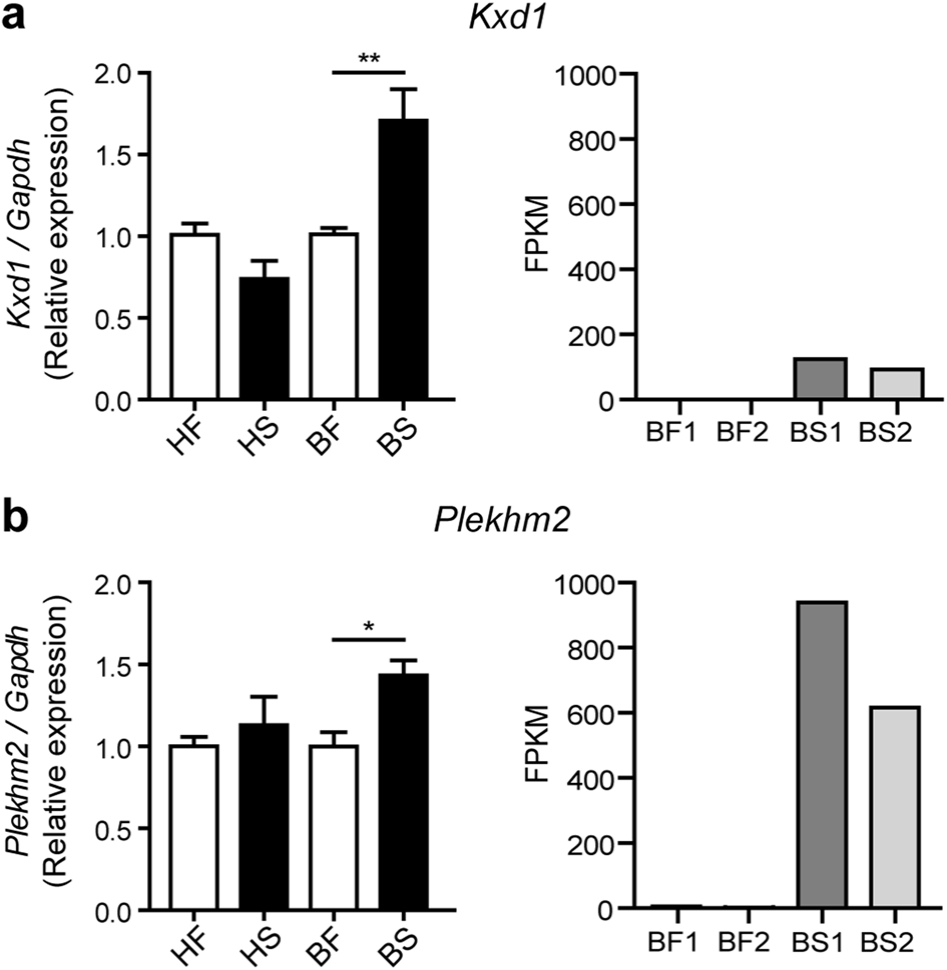
Changes in *Kxd1* and *Plekhm2* expressions validated by qRT-PCR. (**a**) Left, Raw264.7 macrophages were infected with the *M. tuberculosis* reference strain H37Rv or BJN followed by autophagy induction by starvation. *Kxd1* expression was quantified using qRT-PCR. Relative gene expression was normalised to *Gapdh* levels using 2^*−*∆∆ct^ method. Data are means ± SEM from at least three independent experiments; **p < 0.01, relative to the full control set of 1.0 was determined by one-way ANOVA with Tukey’s multiple comparison test. Right, FPKM values of *Kxd1* transcripts from the RNA-Seq results. (**b**) Left, *Plekhm2* expression was quantitated using qRT-PCR as in (**a**). Data are means ± SEM from at least three independent experiments; *p < 0.05, relative to the full control set of 1.0 was determined by one-way ANOVA with Tukey’s multiple comparison test. Right, FPKM values of *Plekhm2* transcripts from RNA-Seq results. *HF* macrophages infected with H37Rv without autophagy induction, *HS* macrophages infected with H37Rv followed by autophagy induction by starvation, *BF* macrophages infected with BJN without autophagy induction, *BS* macrophages infected with BJN followed by autophagy induction by starvation.

Kxd1 and Snapin are components of the BLOC-one-related complex (BORC)^31^ that functions in recruiting Arl8 and together with Plekhm2 links the lysosomes to the microtubule plus-end-directed kinesin motors and moves the lysosomes towards the cell periphery in HeLa cells^31,35^. Nevertheless, while Snapin was reported as a component of the BORC complex described above^31^, it was also shown to be a component of the BLOC-1 complex involved in the biogenesis of lysosome-related organelles such as melanosomes and platelet dense bodies^36^. Previous studies also showed that Snapin functions in recruiting the late endosomes to the microtubule minus-end-directed dynein motor for retrograde trafficking and maturation into lysosomes in neurons^37^. As mention above, our RNA-Seq data showed that *Kxd1*, *Plekhm2* and *Snapin* transcripts were upregulated in RAW264.7 macrophages infected with the BJN strain during autophagy induction by starvation. However, while *Kxd1* and *Plekhm2* were protein-coding transcripts, the *Snapin* transcript was a nonsense-mediated-decay variant (Supplementary Dataset S2). Therefore, we first determined the expression of *Kxd1*, *Plekhm2* and *Snapin* in each condition by qRT-PCR. Results concurred with our RNA-Seq analyses and showed significant increases in the expressions of *Kxd1* and *Plekhm2* transcripts in macrophages infected with the BJN strain subjected to autophagy induction by starvation (Fig. 2a,b). However, when we used primers that could detect all forms of the *Snapin* transcripts, the qRT-PCR results showed a decrease in their expression in the BJN-infected macrophages upon autophagy induction by starvation (Supplementary Fig. S2). Based on these results, we focused our investigation on Kxd1 and Plekhm2 and their roles in resistance of the BJN strain to starvation-induced autophagic control.

### Kxd1 and Plekhm2 are required for evasion of starvation-induced autophagic restriction by the BJN strain

**Figure 3:**
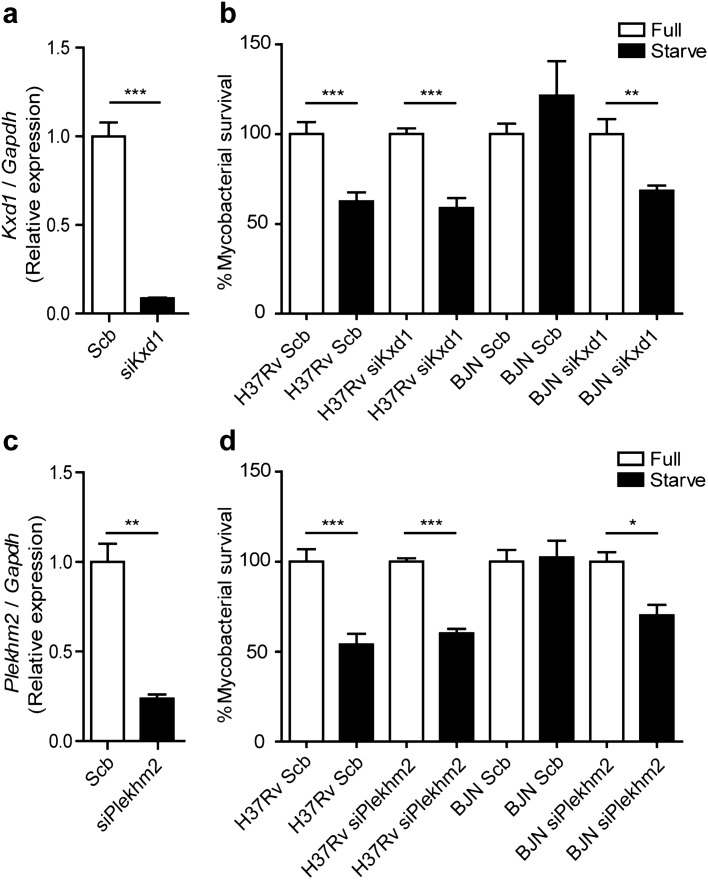
Kxd1 and Plekhm2 required for evasion of starvation-induced autophagic elimination by the BJN strain. (**a**) Raw264.7 cells were transfected with non-targeted scrambled control or *Kxd1*-targeted siRNAs. At 48 h after transfection, *Kxd1* expression levels were determined by qRT-PCR. Data are means ± SEM from at least three independent experiments; ***p < 0.001 was determined by two-tailed unpaired Student’s t-test. (**b**) Kxd1-deficient macrophages were infected with the *M. tuberculosis* reference strain H37Rv or BJN for 1 h and then subjected to autophagy induction by starvation for 4 h. Cells were lysed by osmotic burst to harvest intracellular mycobacteria. Percent mycobacterial survival was determined by plating for CFU. Data are means ± SEM from at least three independent experiments; **p < 0.01 and ***p < 0.001, all relative to the full control set of 100% were determined by one-way ANOVA with Tukey’s multiple comparison test. (**c**) *Plekhm2* expression was depleted using the siRNA-mediated knockdown and expression levels were determined by qRT-PCR as in (**a**). Data are means ± SEM from at least three independent experiments; **p < 0.01 was determined by two-tailed unpaired Student’s t-test. (**d**) Plekhm2-depleted macrophages were infected with different mycobacteria and subjected to autophagy induction by starvation as in (**b**). Mycobacteria intracellular viability was then determined by plating for CFU. Data are means ± SEM from at least three independent experiments; *p < 0.05 and ***p < 0.001, all relative to the full control set of 100% were determined by one-way ANOVA with Tukey’s multiple comparison test.

Based on the above qRT-PCR results and the known function of Kxd1 and Plekhm2, we hypothesised that the BJN strain, by upregulating these genes, escaped starvation-induced autophagic elimination by inducing lysosome relocalisation and hence avoided lysosomal delivery to its phagosome. To determine this, we first depleted the expression of *Kxd1* and *Plekhm2* using the siRNA knockdown technology in RAW264.7 macrophages. Successful knockdown of *Kxd1* and *Plekhm2* expressions were confirmed by qRT-PCR (Fig. 3a,c). A mycobacterial survival assay upon autophagy induction by starvation was then conducted as previously described^38^. In agreement with our previous data^15^, autophagy induction by starvation resulted in restriction of *M. tuberculosis* reference strain H37Rv, while the BJN strain resisted starvation-induced autophagic elimination in scrambled siRNA-treated cells (Fig. 3b,d). Interestingly, in the Kxd1- and Plekhm2-depleted host macrophages, autophagy induction by starvation could now restrict intracellular survival of the BJN strain (Fig. 3b,d). These data indicated that Kxd1 and Plekhm2 are important for the resistance of starvation-induced autophagic control by the BJN strain.

**Figure 4:**
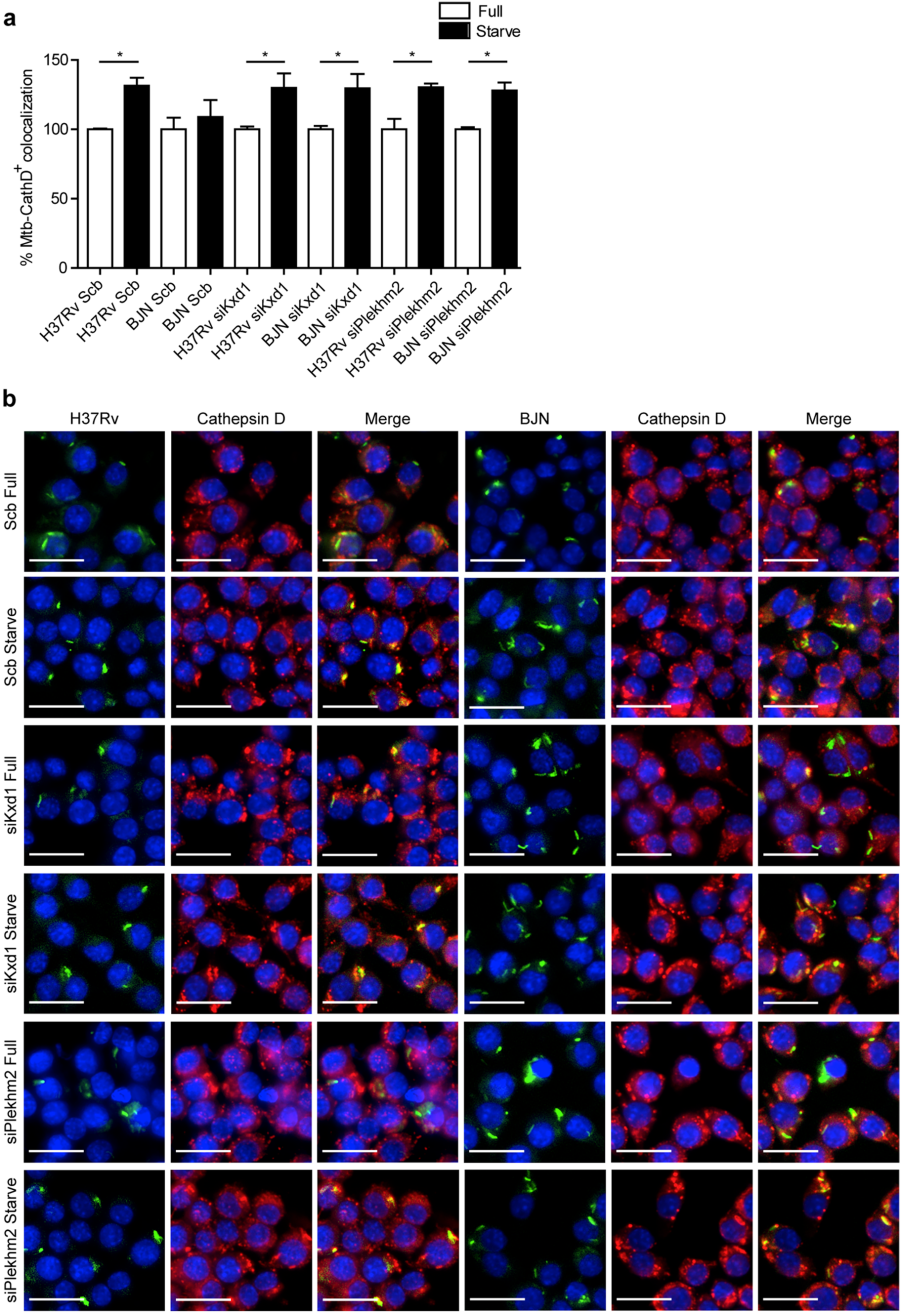
Kxd1 and Plekhm2 reduce lysosomal delivery to BJN phagosomes upon autophagy induction by starvation in RAW264.7 macrophages. (**a**,**b**) Kxd1- and Plekhm2-deficient Raw264.7 macrophages were infected with the Alexa-488-labelled *M. tuberculosis* reference strain H37Rv or BJN for 15 min and chased for 1 h. Host cells were then subjected to autophagy induction by starvation for 2 h. Cells were subsequently fixed and stained for the lysosomal hydrolase Cathepsin D. Percent colocalisation was then analysed by high-content image analysis. Data are means ± SEM from at least three independent experiments; *p < 0.05, all relative to the full control set of 100% was determined by one-way ANOVA with Tukey’s multiple comparison test (**a**). Representative images are shown in (**b**). Bar 20 μm.

To determine whether the reverted resistance phenotype of the BJN strain to starvation-induced autophagic restriction observed above was a result of an increase in lysosomal delivery to the BJN phagosomes, colocalisation of the mycobacteria with Cathepsin D was examined by high-content image analysis in RAW264.7 macrophages. In agreement with our previous report^15^, a significant increase in the colocalisation of *M. tuberculosis* reference strain H37Rv with Cathepsin D upon autophagy induction by starvation was observed in scrambled siRNA-treated cells, while such effect was not seen in control cells infected with the BJN strain^15^ (Fig. 4a,b). However, in Kxd1- and Plekhm2-deficient macrophages, autophagy induction by starvation now resulted in significantly enhanced Cathepsin D colocalisation with BJN phagosomes (Fig. 4a,b). Altogether, these findings suggested that Kxd1 and Plekhm2 play a crucial role in suppressing lysosomal delivery to BJN phagosomes upon autophagy induction by starvation and therefore spare the BJN strain from starvation-induced autophagic restriction.

### The BJN strain dampens lysosome positioning towards the perinuclear region

**Figure 5:**
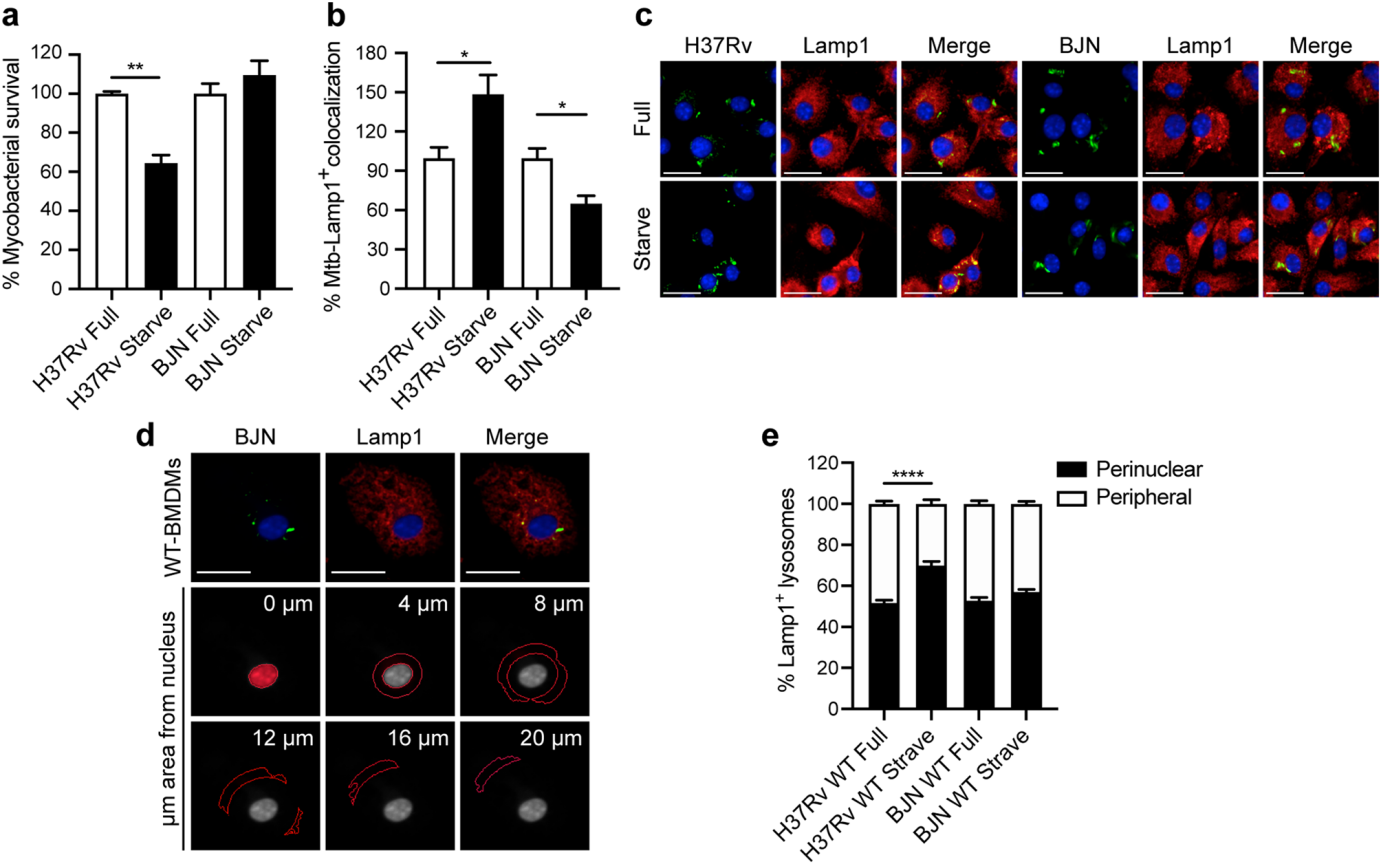
The BJN strain induces lysosome redistribution in BMDMs during autophagy induction by starvation. (**a**) BMDMs were infected with mCherry-expressing H37Rv or BJN for 1 h. Cells were then washed with PBS to remove uninternalised mycobacteria and subjected to autophagy induction by starvation for 4 h. Cells were fixed and the nucleus was stained with Hoechst. The number of intracellular mycobacteria per cell was then determined by high-content image analysis and percent mycobacterial survival was calculated and compared. Data are means ± SEM from at least three independent experiments; **p < 0.01, relative to the full control set of 100% was determined by one-way ANOVA with Tukey’s multiple comparison test. (**b**, **c**) BMDMs were infected with Alexa-546-labelled H37Rv or BJN at MOI of 10 for 15 min and chased for 1 h. Cells were then washed with PBS and subjected to autophagy induction by starvation for 2 h. Samples were then fixed and lysosomes were stained with anti-Lamp1 antibody. The nucleus was stained with Hoechst. Percent mycobacteria-Lamp1 colocalisation was then analysed by high-content image analysis. Data are means ± SEM from at least three independent experiments; *p < 0.05, relative to the full control set of 100% was determined by one-way ANOVA with Tukey’s multiple comparison test (**b**). Representative images are shown in (**c**). (**d**, **e**) BMDMs were infected with Alexa-546-labelled H37Rv or BJN at MOI of 10 for 15 min and chased for 1 h. Cells were then subjected to autophagy induction by starvation and processed for staining with anti-Lamp1 antibody and Hoechst as in (**b**). High-content image analysis was then conducted to count the numbers of Lamp1^+^ lysosomes in each cytoplasmic subarea of the infected cells. A representative image from high-content image analysis is shown in (**d**). Red lines indicate areas at different distances from the nucleus. Percent perinuclear Lamp1^+^ lysosomes (located between 0 and 4 μm from the nucleus) and periphery Lamp1^+^ lysosomes (located between 4 μm from the nucleus and the cell boundary) were then determined and compared. Data are means ± SEM from at least three independent experiments; ****p < 0.0001, relative to the full control was determined by one-way ANOVA with Tukey’s multiple comparison test (**e**). Mycobacteria were pseudocoloured green. Bar 20 μm.

During autophagy induction by starvation, autophagosomes engulf cytosolic substrates and deliver them to the acidic lysosomes concentrating at the perinuclear region, resulting in degradation of the sequestered contents^22^. Starvation also induces positioning of the peripheral lysosomes along microtubules towards the perinuclear region for fusion with autophagosomes^39^. Kxd1 and Plekhm2 were previously shown to function in lysosomal transport along microtubules towards the cell periphery^31,35,40^. As our results showed that BJN-infected RAW264.7 macrophages induced the expression of these genes during autophagy induction by starvation, we thus examined the position of lysosomes in these cells. The size of RAW264.7 macrophages used in our study was relatively small compared to the HeLa cells and neurons previously used in lysosome positioning analysis^31,35,41^; therefore, we used primary bone marrow-derived macrophages (BMDMs) to determine the location of lysosomes in BJN-infected cells. In agreement with our previous data for RAW264.7 macrophages^15^, induction of autophagy by starvation in BMDMs resulted in restriction of the *M. tuberculosis* reference strain H37Rv, while BJN resisted starvation-induced autophagic elimination (Fig. 5a). Enhanced colocalisation of H37Rv phagosomes with Lamp1, used as a marker for lysosomes, was also shown upon autophagy induction by starvation (Fig. 5b,c). By contrast, we observed a significant decrease in BJN phagosome-Lamp1 colocalisation upon autophagy induction by starvation (Fig. 5b,c). We then determined the lysosome position in cells infected with different mycobacteria, using high-content image analysis, to quantify the numbers of lysosomes distributed in each subarea of the infected cells. Figure 5d shows the representative image from high-content image analysis of Lamp1^+^ lysosomes. The boundary of each infected cell and its nucleus was first determined. From this, the cytoplasmic area was subdivided into 0, 4, 8, 12, 16 and more than 20 μm distance from the nucleus. Lysosome numbers in each subarea were then quantitated and the sum was set at 100%. Percent perinuclear Lamp1^+^ lysosomes (located between 0 and 4 μm distance from the nucleus) and percent peripheral Lamp1^+^ lysosomes (located between 4 μm distance from the nucleus and the cell boundary) were then calculated. Significantly enhanced lysosome positioning towards the perinuclear region was observed upon autophagy induction by starvation in H37Rv-infected BMDMs but such an effect was not observed in cells infected with BJN (Fig. 5e). These data indicated that the BJN strain reduced lysosome relocation towards the perinuclear region during autophagy induction by starvation in BMDMs.

### Kxd1 and Plekhm2 facilitate the inhibition of lysosome repositioning towards the perinuclear region by the BJN strain

**Figure 6:**
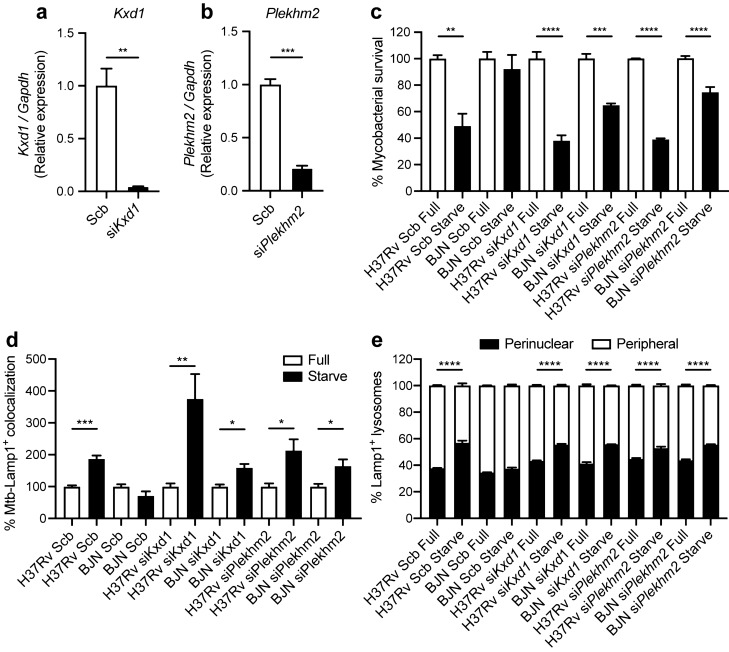
Kxd1 and Plekhm2 are required for suppression of lysosome redistribution towards the perinuclear region upon autophagy induction by starvation in BJN-infected BMDMs. (**a**,**b**) BMDMs were transfected with the control scrambled siRNAs or siRNAs targeting *Kxd1* and *Plekhm2*. At 48 h after transfection, *Kxd1* and *Pleckhm2* expression levels were determined by qRT-PCR. Data are means ± SEM from at least three independent experiments; **p < 0.01 and ***p < 0.001 were determined by two-tailed unpaired Student’s t-test. (**c**) Kxd1- and Pleckhm2-deficient BMDMs were infected with mCherry-expressing H37Rv or BJN for 1 h. Cells were then washed with PBS and subjected to autophagy induction by starvation for 4 h. Cells were fixed and the nucleus was stained with Hoechst. High-content image analysis was then conducted to determine the number of intracellular mycobacteria per cell. Percent mycobacterial survival was calculated and then compared. Data are means ± SEM from at least three independent experiments; **p < 0.01, ***p < 0.001 and ****p < 0.0001, all relative to the full control set of 100%, were determined by one-way ANOVA with Tukey’s multiple comparison test. (**d**) Kxd1- and Plekhm2-deficient BMDMs were infected with Alexa-546-labelled H37Rv or BJN at MOI of 10 for 15 min and chased for 1 h. Cells were then washed with PBS and subjected to autophagy induction by starvation for 2 h. Samples were then fixed and processed for staining with anti-Lamp1 antibody and Hoechst. Percent mycobacteria-Lamp1 colocalisation was then analysed by high-content image analysis. Data are means ± SEM from at least three independent experiments; *p < 0.05, **p < 0.001 and ***p < 0.0001, all relative to the full control set of 100% were determined by one-way ANOVA with Tukey’s multiple comparison test. (**e**) Kxd1- and Plekhm2-deficient BMDMs were infected with Alexa-546-labelled H37Rv or BJN at MOI of 10 for 15 min and chased for 1 h. Cells were then subjected to autophagy induction by starvation for 24 h and processed for staining with anti-Lamp1 antibody and Hoechst. High-content image analysis was then conducted to count the numbers of Lamp1^+^ lysosomes in each cytoplasmic subarea of the infected cells. Percent perinuclear Lamp1^+^ lysosomes and periphery Lamp1^+^ lysosomes were then calculated and compared as in Fig. 5. Data are means ± SEM from at least three independent experiments; ****p < 0.0001, relative to the full control was determined by one-way ANOVA with Tukey’s multiple comparison test.

Next, we determined the importance of Kxd1 and Plekhm2 in dampening lysosome relocation towards the perinuclear region by the BJN strain during autophagy induction by starvation in BMDMs. We conducted the siRNA-mediated knockdown of *Kxd1* and *Plekhm2* expressions in BMDMs. Successful knockdown was confirmed by qRT-PCR (Fig. 6a,b). Similar to our data in RAW264.7 macrophages, autophagy induction by starvation resulted in enhanced killing of H37Rv, while the BJN strain resisted starvation-induced autophagic elimination in scrambled siRNA-treated BMDMs (Fig. 6c). However, upon depletion of *Kxd1* and *Plekhm2* expressions in BMDMs, autophagy induction by starvation now restricted intracellular survival of the BJN strain (Fig. 6c). We also determined lysosomal delivery to *M. tuberculosis* phagosomes in these cells. In agreement with our results for RAW264.7 macrophages, we observed a significant increase in the colocalisation of H37Rv with lysosomes, marked by Lamp1, in scrambled siRNA-treated BMDMs upon autophagy induction by starvation, while such an effect was not observed in control cells infected with BJN (Fig. 6d). However, in Kxd1- and Plekhm2-deficient macrophages, autophagy induction by starvation now significantly enhanced Lamp1 colocalisation with BJN phagosomes (Fig. 6d).

We then determined the lysosome position in the aforementioned cells. While a significant increase was shown in lysosome positioning towards the perinuclear region upon autophagy induction by starvation in scrambled siRNA-treated H37Rv-infected BMDMs, such an effect was not observed in control cells infected with BJN (Fig. 6e). Upon Kxd1 and Pleckhm2 depletion, increased lysosome relocation towards the perinuclear region upon autophagy induction by starvation was seen in BJN-infected BMDMs (Fig. 6e). This result was also confirmed in *Plekhm2*^*−/−*^ Hoxb8 progenitor cells differentiated into macrophages^42–45^ (Supplementary Fig. S3). In wild-type Hoxb8 macrophages infected with H37Rv, autophagy induction by starvation resulted in an increase in lysosome relocation towards the perinuclear region, while such an effect was not observed in BJN-infected cells (Supplementary Fig. S3). In the *Plekhm2*^*−/−*^ Hoxb8 macrophages, autophagy induction by starvation now induced relocation of lysosomes towards the perinuclear region in the BJN-infected cells (Supplementary Fig. S3). Altogether, these findings suggested that Kxd1 and Plekhm2 are important for the suppression of lysosomal positioning towards the perinuclear region and delivery to BJN phagosomes upon autophagy induction by starvation, therefore sparing the BJN strain from starvation-induced autophagic restriction.

## Discussion

Tuberculosis is a major public health problem, with the recent emergence of multidrug-resistant infections. The identification of new drugs with novel mechanisms is therefore urgently needed. Autophagy has been demonstrated to play a key role in cell autonomous immunity against *M. tuberculosis* in host macrophages^23–29,46–51^. Induction of autophagy leads to enhanced mycobacterial phagosome acquisition of lysosomal hydrolases, resulting in the digestion of intracellular *M. tuberculosis* reference strains such as H37Rv and strains belonging to the East African Indian genotype^15,25,27,28,38^. However, strains belonging to the notorious *M. tuberculosis* Beijing genotype were demonstrated to have a special ability to resist starvation-induced autophagic elimination, with factors involved remaining unclear^15^.

**Figure 7:**
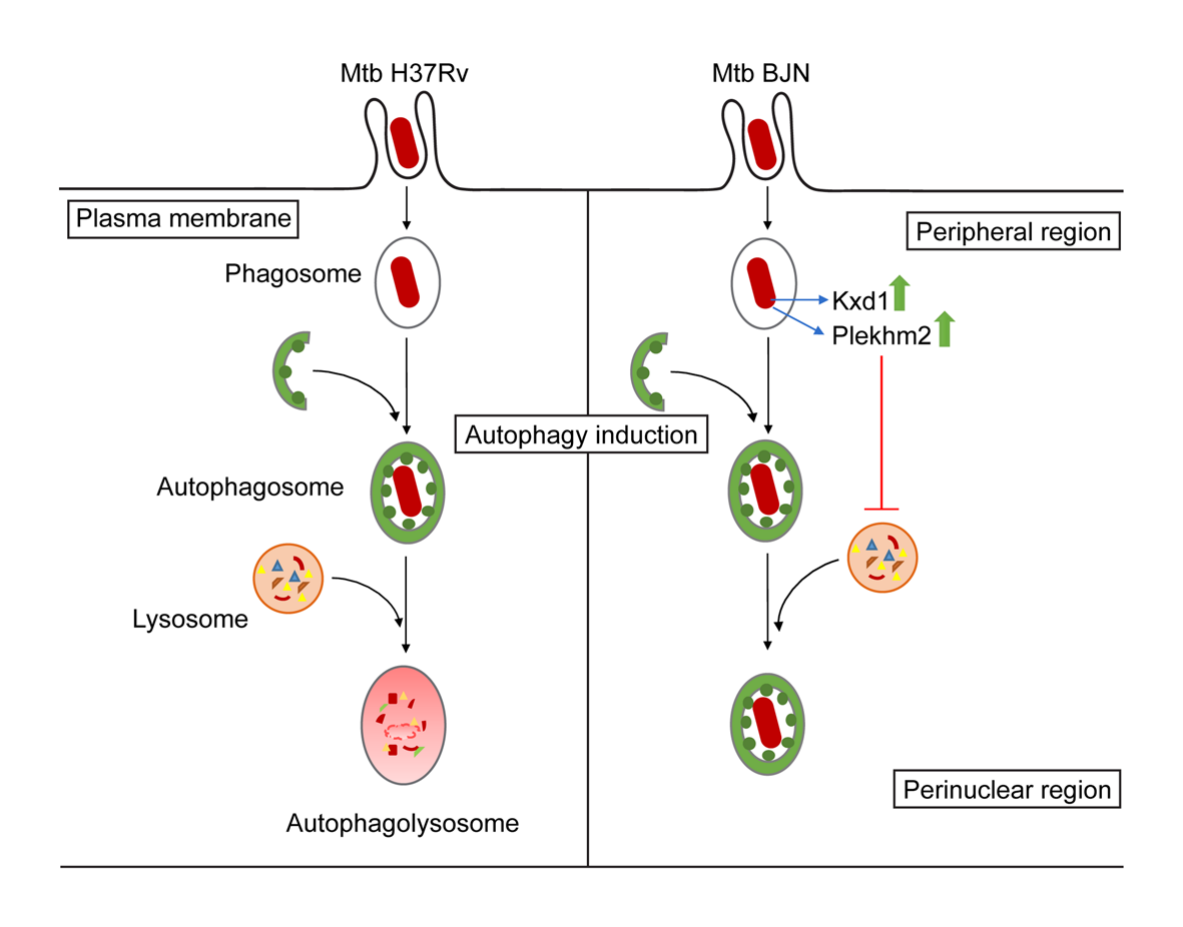
Schematic representation of induced lysosome repositioning as an escape mechanism from starvation-induced autophagic restriction by the BJN strain. Autophagy induction by starvation promotes *M. tuberculosis* reference strain H37Rv elimination in the autophagolysosome. The BJN strain, on the other hand, can upregulate the expression of Kxd1 and Plekhm2 resulting in the suppression of lysosome positioning towards the perinuclear region during autophagy induction by starvation, thus sparing its phagosome from autophagy-mediated lysosomal delivery and destruction.

In this study, we utilised RNA-Seq technology to pinpoint factors responsible for evasion of starvation-induced autophagic restriction by the autophagy-resistant Beijing strain (BJN). Our results identified several unique genes that were differentially regulated in BJN-infected macrophages subjected to autophagy induction by starvation (Fig. 1, Table 1 and Supplementary Dataset S1), with findings confirmed by qRT-PCR (Supplementary Fig. S1 and Fig. 2). In silico GO analysis of differentially regulated genes revealed several altered and enriched pathways upregulated in host cells infected with the BJN strain upon autophagy induction by starvation, including the lysosome localisation pathway (Table 2). Depletion of the expression of *Kxd1* and *Plekhm2*, two of the genes that function in the above pathway, reverted the resistance phenotype of the BJN strain to starvation-induced autophagic restriction (Fig. 3). High-content image analysis showed a block in lysosomal delivery to BJN phagosomes dependent on Kxd1 and Plekhm2 during autophagy induction by starvation (Fig. 4). The BJN strain was also shown to suppress lysosome relocation towards the host cell perinuclear region in host macrophages subjected to autophagy induction by starvation, depending on Kxd1 and Plekhm2 (Figs. 5 and 6). Taken together, our findings concurred with a model in which the *M. tuberculosis* BJN strain evaded starvation-induced autophagic restriction by upregulating gene functions in lysosome positioning towards the cell periphery, resulting in suppression of lysosome redistribution towards the host cell perinuclear region upon autophagy induction by starvation, therefore sparing the mycobacteria from lysosomal delivery and destruction by lysosomal hydrolases (Fig. 7). Thus, a new strategy for autophagy evasion by a pathogen was identified.

The importance of autophagy in immune defence against intracellular pathogens was substantially supported by studies showing that many intracellular pathogens employed various mechanisms to evade autophagy^16^. These mechanisms included the targeting of key autophagy proteins required for autophagosome formation such as Beclin-1 and LC3^52–56^, concealing molecular tags to prevent pathogen detection by autophagy receptors^57–59^, blocking the maturation of autophagosomes using anti-maturation factors^60,61^ and inhibiting autolysosome acidification and degradation of the engulfed contents by perforation of the membrane^62,63^. On the other hand, a few pathogens adapted to survive and replicate in acidified autophagosomes/autolysosomes^64,65^. In this study, we identified lysosome repositioning as a new mechanism for autophagy escape by *M. tuberculosis*. To the best of our knowledge, inducing lysosome redistribution as a way to avoid autophagy-mediated elimination by a pathogen has not been previously reported. Although lysosome positioning has been shown to be modulated by *Salmonella* Typhimurium and Hepatitis C virus (HCV), these pathogens did so to promote their spread from host cells^66,67^.

In the case of *S.* Typhimurium, the bacterium recruited the plus-end-directed kinesin-1 motor to its compartment, the *Salmonella*-containing vacuole, resulting in anterograde movement of the vacuole towards the host cell periphery to promote bacterial dissemination^67,68^. Recruitment of kinesin-1 to the *Salmonella*-containing vacuole was shown to depend upon Arl8 and Plekhm2^67,68^. Similarly, HCV modulated lysosome repositioning by cleaving RILP, the protein responsible for linking Rab7-containing lysosomes to the minus-end-directed dynein motor. This redirected the Rab7-containing vesicles to the plus-end-directed kinesin motor for anterograde movement towards the plasma membrane to promote virion secretion^66^. HCV has been shown to exploit autophagy and enhance virus production; it was also shown that redirecting lysosomes towards the host cell periphery inhibited the overall autophagic flux in HCV-infected cells^69^. Therefore, HCV and *S.* Typhimurium appeared to recruit the kinesin motors to their compartments to facilitate their anterograde trafficking towards the plasma membrane and promote cell-to-cell spread. We also showed that Kxd1 and Plekhm2 are required for evasion of starvation-induced autophagic elimination by the autophagy-resistant Beijing strain. However, the involvement of Arl8 and kinesin motors in this process remains undetermined and warrants further investigation. Our RNA-Seq data also indicated that both *Kxd1* and *Plekhm2* were uniquely upregulated in host macrophages infected with the autophagy-resistant Beijing strain during autophagy induction by starvation. How the Beijing strain upregulates the expression of *Kxd1* and *Plekhm2* remains unclear. *M. tuberculosis* possesses several secretion systems that can deliver mycobacterial components and virulence factors into host cells. Involvement of such factors in ensuing the increased expression of *Kxd1* and *Plekhm2* is suspected but requires further study.

Kxd1 is a component of the BORC complex^31^ that is composed of eight proteins including BLOS1, BLOS2, Snapin, Kxd1, Myrlysin, Lyspersin, Diaskedin and MEF2BNB. The first three proteins are also components of the BLOC-1 complex involved in the biogenesis of lysosome-related organelles^31^. Previous studies showed that the BORC complex functions in recruiting Arl8 and together with Plekhm2 links the lysosomes to the microtubule plus-end-directed kinesin motors, thereby moving them towards the periphery in HeLa cells^31,35^. Our results showed that Kxd1 and Plekhm2 are required for evasion of starvation-induced autophagic elimination by the autophagy-resistant Beijing strain but whether other proteins in the BORC complex are also involved in this process remains to be examined. Our RNA-Seq data showed that the autophagy-resistant Beijing strain also upregulated the *Snapin* transcript as a nonsense-mediated-decay variant. Further investigation by qRT-PCR analysis to detect all forms of *Snapin* transcripts confirmed overall decreased expression of *Snapin* in BJN-infected macrophages during autophagy induction by starvation. Snapin is a component of both BORC^31^ and BLOC-1 complexes^36^. Previous studies showed that Snapin functions as an adaptor to link late endosomes to the microtubule minus-end-directed dynein motor for retrograde movement and maturation into lysosomes in neurons^37^. A decrease in *Snapin* expression may contribute to the autophagy-resistant phenotype of the BJN strain by dampening the maturation of lysosomes in the host macrophages. Our data indicated that during autophagy induction by starvation, around 50% of Lamp1^+^ lysosomes in the BJN-infected macrophages were located at the peripheral region. Whether these were immature lysosomes was not determined. Consistent with this idea, an accumulation of immature lysosomes was observed in *Snapin*^*−/−*^ neurons^37^. Further investigation is required to determine whether decreased *Snapin* expression would result in reduced mature lysosomes and contribute to the resistance of the BJN strain to starvation-induced autophagic restriction.

The position of the lysosome in the cytoplasm is known to affect its functions. As mentioned above, lysosomes can move bidirectionally on the microtubule and retrograde trafficking towards the cell centre or anterograde trafficking towards the cell periphery^70^. Lysosome positioning can also be regulated by various conditions such as acidic cytoplasmic and extracellular pH, which induce the outward movement of lysosomes, while starvation induces the inward movement of lysosomes causing juxtanuclear clustering^71^. It is known that upon autophagy induction by starvation, autophagosomes move towards the cell centre in a dynein-dependent manner along microtubule tracks to fuse with the juxtanuclear lysosomes^72,73^. These findings are consistent with our model in which the autophagy-resistant Beijing strain, by upregulating lysosome redistribution towards the cell periphery and away from the juxtanuclear region, escaped from starvation-induced lysosomal delivery and thus elimination. Interestingly, a recent paper also showed that juxtanuclear lysosomes are more acidic and have higher Cathepsin L activity than peripheral lysosomes^74^. Whether peripheral lysosomes in BJN-infected macrophages during autophagy induction by starvation are less acidic and possess decreased Cathepsin activity is not known and warrants further study.

Our findings identified the importance of lysosome repositioning as a new autophagy evasion strategy employed by the *M. tuberculosis* Beijing strain. This pathway provided a new target for drug discovery against this disease. Interestingly, a recent study screened small molecule libraries and identified kinesore, a compound that inhibits Plekhm2-kinesin interaction, while concurrently activating kinesin-1 activity^75^. HeLa cells treated with kinesore also showed accumulation of lysosomes in the juxtanuclear region^75^. Plekhm2 was identified in our study as important for lysosome redistribution in BJN-infected macrophages during autophagy induction by starvation and required for escape of starvation-induced autophagic elimination. Therefore, it would be interesting to determine whether kinesore can revert the autophagy evasion phenotype of the Beijing strain. The occurrence of kinesore also serves as a proof-of-concept that this pathway is targetable and may provide passage for new drugs with novel mechanisms needed to fight against this emerging disease.

## Materials and methods

### Cells and bacterial culture

Raw264.7 macrophages (ATCC) were cultured in Dubecco’s modified Eagle’s medium (DMEM; Gibco) supplemented with 10% fetal bovine serum (FBS; Gibco), 0.37% sodium bicarbonate (Sigma) and 4 mM l-glutamine (Hyclone) (full medium) at 37 °C and 5% CO_2_. BMDMs were prepared from freshly isolated bone marrow cells of C57/BL6 mice (Nomura Siam International, Thailand) using L929 condition media as described elsewhere with modification^76^. BMDMs were stored in liqiuid nitrogen until use. All animal procedures were approved by the Institutional Animal Care and Use Committee of Faculty of Medicine, Chulalongkorn University (approval protocol No. 025/2562) and were performed in compliance with the ARRIVE guidelines. For experiments, cryopreserved BMDMs were thawed and then cultured in Dulbecco's Modified Eagle's Medium (DMEM, Gibco) supplemented with 10% fetal bovine serum (FBS, Gibco), 1% sodium pyruvate (Sigma), 1% HEPES (Gibco) and 20% L929 cell conditioned medium. Earle’s Balanced Salt Solution (EBSS; Gibco) (starve medium) was used for autophagy induction. *M. tuberculosis* reference strain H37Rv (ATCC) and the autophagy-resistant Beijing strain (BJN)^15^ were cultured in Middlebrook 7H9 medium or on 7H10 agar containing 10% oleic acid-albumin-dextrose-catalase (OADC; BD), 0.2% glycerol and 0.05% Tween 80 at 37 °C. Before experiments, log-phase cultures were collected, washed twice with PBS, and resuspended in complete medium and homogenised to generate single-cell suspension before measuring the absorbance at 600 nm. mCherry-expressing H37Rv or BJN were generated by electroporating the mycobacteria with 4.6 µg of pCHERRY3 plasmid^77^ (Addgene, plasmid# 24659) as previously described^78^. Transformants were selected with hygromycin (100 µg/mL; Invitrogen) and cultured as described above.

### Fluorescent dye, antibodies and siRNAs

For immunofluorescence assays, monoclonal antibody against Cathepsin D (R&D Systems) was used at 1:50 and monoclonal antibody against Lamp1 (DSHB) was used at 1:25. The fluorescent dye Hoechst 33342 (Thermo Fisher Scientific) was used at 1:500 and secondary antibodies (Thermo Fisher Scientific) were used at 1:400. All siRNAs used in this study were from Dharmacon.

### *M. tuberculosis* infection of RAW264.7 macrophages and total RNA isolation

Raw264.7 macrophages were grown in 75 cm^3^ tissue culture flask at 80% confluency. Cells were then infected with *M. tuberculosis* reference strain H37Rv or BJN at MOI of 10 for 2 h. After incubation, cells were washed with PBS three times and subjected to autophagy induction by starvation for 2 h. Total RNAs were collected using Trizol. Briefly, the media were removed and 2 mL of TRI Reagent (Thermo Fisher Scientific) were added and incubated for 5 min at room temperature. The samples were transferred to new 1.5 mL RNase-free tubes and centrifuged at 13,000 rpm for 1 min. The supernatants were transferred to new 1.5 mL RNase-free tubes and nucleic acids were isolated from each sample by the chloroform extraction method. Total RNAs were then isolated using the Ambion RiboPure (Thermo Fisher Scientific) according to the manufacturer’s instruction. Genomic DNAs were digested using DNase I (Thermo Fisher Scientific).

### Illumina library construction

The ratio of optical density (OD) at 260 nm and 280 nm was used to assess the purity of total RNAs using DeNovix fluorometer (DeNovix). RNA integrity number (RIN) > 7.0 was obtained from each sample condition using Agilent 2100 Bioanalyzer (Agilent). Approximately 500 ng of the total RNAs from each sample were used to synthesize the cDNAs and create individually indexed strand-specific RNA-Seq libraries using Truseq stranded mRNA library preparation kit (Illumina). Briefly, magnetic oligo (dT) beads captured mRNA molecules containing the poly-A and directed to cDNA synthesis. Then, AmPure XP beads (Beckman Coulter) were used to purified cDNAs from the reaction mix. Subsequently, indexing adaptors were ligated to cDNAs, then the quality and quantity of all cDNA libraries were checked using Agilent 2100 Bioanalyzer and DeNovix fluorometer, respectively. The indexed cDNA libraries were pooled in the equimolar quantity and loaded into flow cell for cluster generation and paired-end 2 × 75 nucleotide read sequencing on the Illunina NextSeq 500 sequencer. The sequencing process was carried out at Omics Sciences and Bioinformatics Center, Chulalongkorn University, Bangkok, Thailand.

### Differential expression analyses of RNA-Seq data

The newly identified sequence reads were obtained and subjected to the bioinformatics analyses. Raw read data files were subjected to quality control using FASTQC software. Adapter and poor quality reads were removed using Trimmomatic^79^. The filtered reads were aligned to a mouse reference genome (GRCm38.p6) using TopHat2 aligner software^80^. StringTie^81^ and prepDE.py script were used to assemble transcripts from RNA-Seq reads that have been aligned to the genome, reconstruct all the isoforms expressed from each gene, and estimate the relative abundance of those isoforms. Subsequently, differentially expressed isoforms were identified with FDR < 0.05 using edgeR^82,83^. Gene ontology and pathway enrichment analyses were performed using the differentially expressed transcripts on the web-based bioinformatics tool DAVID 6.8 (https://david.ncifcrf.gov/).

### RNA-Seq validation and qRT-PCR

Total RNAs were isolated from Raw264.7 macrophages and BMDMs infected with or without different mycobacteria subjected to autophagy induction by starvation as described above. Five hundred nanograms of total RNAs were used in reverse transcription using random hexamers (Promega). Primers used for the target genes were generated commercially (Ward Medic; Supplementary Table S2). The resulted cDNAs were used as the reaction templates for qRT-PCR analyses. In brief, qRT-PCR analyses were conducted using the thermo cycler (Rotor-Gene Q, Qiagen) with HotStarTaq DNA polymerase (Qiagen), 0.1 mM forward and reverse primers, 4 mM MgCl_2_, dNTPs (Promega) and SYBR green (Invitrogen) at various annealing temperatures (54–66 °C). Reverse transcriptase-minus templates were used as negative controls. Melting curve analyses were done to verify the specificity of the PCR products. All threshold signals obtained were analysed to be > 95% efficient. The optimal condition for each target gene was selected and the amplification products were analysed using the Q-Rex software version 1.0.1. Signals were normalised to the housekeeping *Gapdh* transcript. The results were presented as relative quantification using 2^*−*∆∆ct^ method.

### siRNA-mediated knockdown

siRNA-mediated knockdown was performed as previously described^38^. In brief, Raw264.7 macrophages or BMDMs were harvested and re-suspended in 90 µL of solution V (for RAW264.7 cells; Lonza) or solution for mouse macrophages (for BMDMs; Lonza). Scrambled siRNAs or siRNAs against *Kxd1* or *Plekhm2* (Dhamacon; 1.5 µg/reaction) were added to the cell suspension, transferred to an electroporation cuvette and nucleofected using the Amaxa Nucleofector apparatus (Amaxa Biosystems) with program D-032 (for RAW264.7 macrophages) or Y-001 (for BMDMs). At 24 h after transfection, cells were harvested and plated for assays.

### Mycobacterial survival assay

Mycobacterial survival assay by CFU analysis was conducted as previously described^38^. In brief, Raw264.7 macrophages were plated into 12-well plates (3 × 10^5^ cells per well). Cells were infected with different strains of *M. tuberculosis* at MOI of 10 for 1 h, washed with PBS to remove the uninternalised mycobacteria and incubated for 4 h with full or starvation medium. Cells were then lysed by osmotic burst to harvest the intracellular mycobacteria followed by serial dilution and plated onto the Middlebrook 7H10 agars. Plates were incubated for 14–20 days at 37 °C and colonies were counted. Percent mycobacterial survival was then determined and compared between conditions.

Mycobacterial survival assay by high-content image analysis was conducted by infecting BMDMs (2.5 × 10^4^ cells per well) in 96-well black plates with the mCherry-expressing H37Rv or BJN at MOI of 10 for 1 h, washed with PBS to remove the uninternalised mycobacteria and incubated for 4 h with full or starvation medium. Cells were fixed with 4% paraformaldehyde and the nucleus was stained with Hoechst for 15 min. Samples were then subjected to high-content image analysis (Operetta, PerkinElmer) to count the number of intracellular mycobacteria per cell. Percent mycobacterial survival was then calculated and compared between conditions.

### High-content image analysis for lysosomal colocalisation with mycobacteria and lysosome distribution

To determine the colocalisation of mycobacteria with lysosomal markers, RAW264.7 macrophages or BMDMs were plated into 96-well black plates (2.5 × 10^4^ cells per well). At 48 h post transfection, cells were infected with *M. tuberculosis* labeled with Alexa-488 (for RAW264.7 macrophages) or Alexa-546 (for BMDMs) at MOI of 10 for 15 min, washed with PBS to remove the uninternalised mycobacteria and chased for 1 h as previously described^38^. Cells were then subjected to autophagy induction by starvation for 2 h. Cells were fixed with 4% paraformaldehyde and processed for Cathepsin D staining (for RAW264.7 macrophages) or Lamp1 staining (for BMDMs). The nucleus was stained with Hoechst for 15 min. Samples were then subjected to high-content image analysis (Operetta, PerkinElmer) for percent mycobacteria-lysosomal marker colocalisation.

To determine the lysosome distribution, BMDMs were plated into 96-well black plates (2.5 × 10^4^ cells per well). After 24 h, cells were infected with Alexa-546-labeled *M. tuberculosis* H37Rv or BJN at MOI of 10 for 15 min, washed with PBS to remove the uninternalised mycobacteria and chased for 1 h as described above. Cells were subjected to autophagy induction by starvation for 24 h, fixed with 4% paraformaldehyde and processed for Lamp1 and Hoechst staining. High-content image analysis (Operetta, PerkinElmer) was then conducted to count the number of Lamp1^+^ lysosomes in each cytoplasmic subarea of the infected cells. The different cytoplasmic subarea was defined by the distance from the nucleus. Percent perinuclear Lamp1^+^ lysosomes (located between 0 and 4 μm distance from the nucleus) and percent peripheral Lamp1^+^ lysosomes (located between 4 μm distance from the nucleus and the cell boundary) were then determined and compared between conditions.

### Statistical analysis

Unless otherwise stated, all experiments were conducted at least three times and the data were pooled for determination of the mean ± standard error of the mean (S.E.M.). All data were analysed by the Prism software (GraphPad) using two-tailed unpaired Student’s t-test or one-way ANOVA. A p-value less than 0.05 was considered to indicate statistical significance.

## Supplementary Information

Supplementary Information 1.

Supplementary Information 2.

Supplementary Information 3.

## Data Availability

The RNA-Seq data have been uploaded to the Gene Expression Omnibus (GEO) database (Accession number GSE151633).
