# Supplementary material for: Lysosome repositioning as an autophagy escape mechanism by Mycobacterium tuberculosis Beijing strain
Source: Sci Rep. 2021 Feb 22;11:4342. doi: 10.1038/s41598-021-83835-4 (PMC7900199; doi:10.1038/s41598-021-83835-4)

Supplementary Information

Lysosome repositioning as an autophagy escape mechanism by *Mycobacterium tuberuculosis* Beijing strain

Thanida Laopanupong[1], Pinidphon Prombutara[2,3], Phongthon Kanjanasirirat[4], Salisa Benjaskulluecha[5], Atsadang Boonmee[6], Tanapat Palaga[5,6], Stephane Méresse[7], Jiraporn Paha[1], Tegar Adriansyah Putra Siregar[1], Tanawadee Khumpanied[4], Suparerk Borwornpinyo[4,8], Angkana Chaiprasert[9,10], Pongsak Utaisincharoen[1] & Marisa Ponpuak[1,11*]

Supplementary Materials and Methods

Hoxb8 culture

Wild-type and *Plekhm2*$^{-/-}$ knockout Hoxb8 progenitor cells were grown in the progenitor outgrowth medium containing RPMI1640 (Gibco) supplemented with 10% fetal calf serum (Gibco), 50 µM 2-mercaptoethanol (Gibco), 4 mM L-glutamine (Hyclone), 1% MEM (Gibco), 5% GM-CSF conditioned medium and 1 µM Beta-estradiol (Sigma). The progenitors were then differentiated into macrophages in Macro-φ differentiation medium containing RPMI1640 (Gibco) supplemented with 10% fetal calf serum (Gibco), 50 µM 2-mercaptoethanol (Gibco), 4 mM L-glutamine (Hyclone), 1% MEM (Gibco) and 10% M-CSF conditioned medium.

SDS-PAGE and immunoblotting

To determine the expression of Plekhm2 in wild-type and *Plekhm2*$^{-/-}$ knockout Hoxb8 cells, they were lysed by the addition of 20 µL/cm^2 of lysis buffer (PBS, 1% NP-40, 1 mM PMSF, pH 7). Samples were centrifuged at 13,000 rpm in a table-top centrifuge. Supernatants were saved, treated with 5x Laemmli buffer, run on 8% SDS-PAGE gels and transferred onto Immobilon-P membranes (Millipore). Blocking, incubation with antibodies, and washing were performed in PBS with 4% dry milk and 0.05% Tween-20. Polyclonal antibodies against Plekhm2 and monoclonal antibody against Actin were used at 1:1000. Detection was performed using a chemiluminescence system.

High-content image analysis for lysosome distribution in Hoxb8 cells

Wild-type and *Plekhm2*$^{-/-}$ knockout Hoxb8 progenitor cells were differentiated into macrophages as described above for 8 days. Cells were then infected with the Alexa-488-labeled *M. tuberculosis* reference strain H37Rv or BJN for 15 min and chased for 1 hr. Host macrophages were then subjected to autophagy induction by starvation for 24 hr. Cells were fixed and stained for anti-Lamp1 antibody and Hoechst. The number of lysosomes located in each subarea of the infected cells was then determined by high-content image analysis (Operetta, PerkinElmer). Percent perinuclear Lamp1$^+$ lysosomes (located between 0 and 4 µm

distance from the nucleus) and percent peripheral Lamp1^{+} lysosomes (located between 4 μm distance from the nucleus and the cell boundary) were then determined as described in the Materials and Methods.

Supplementary Figures

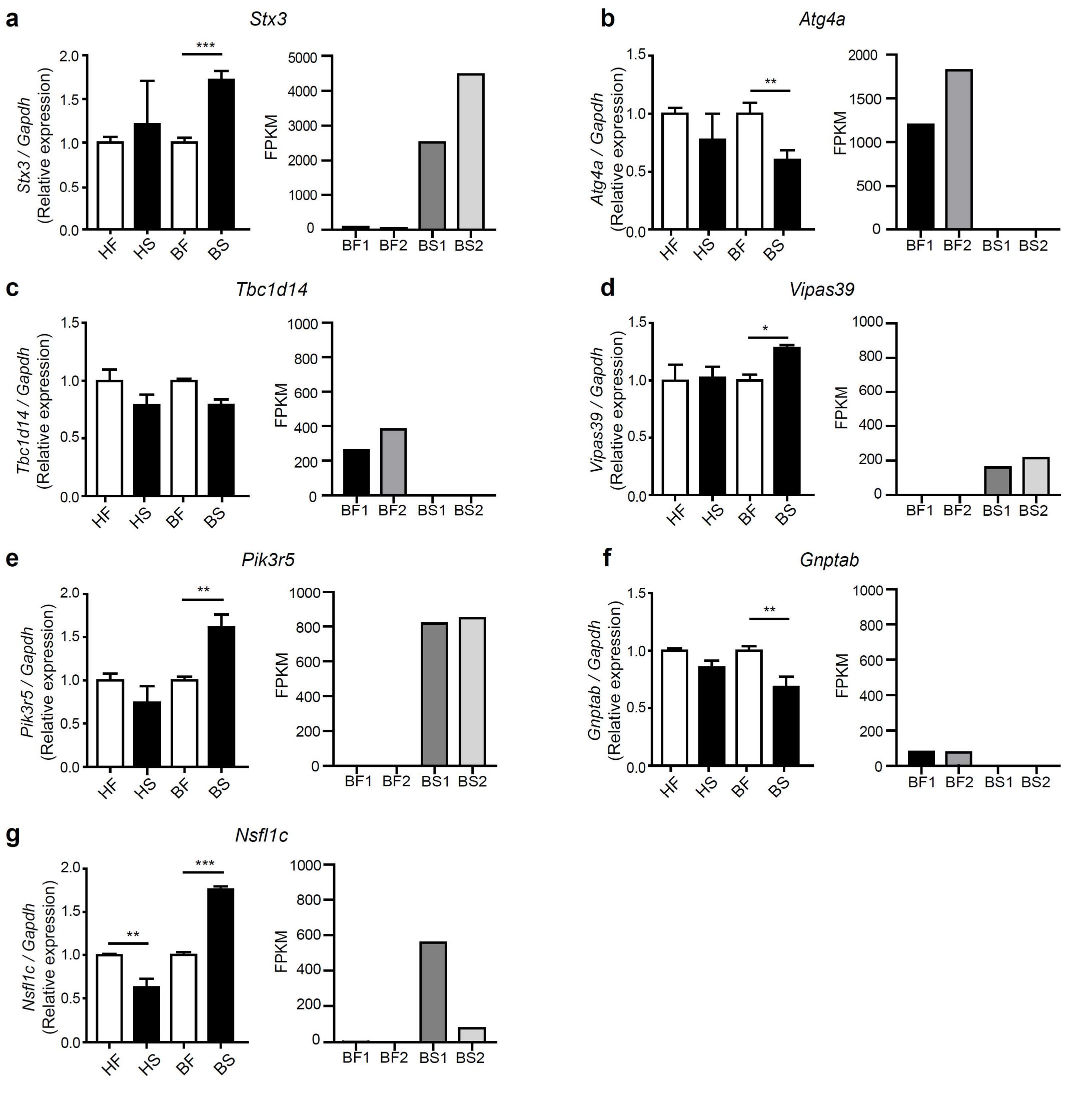

Supplementary Figure S1. Validation of RNA-Seq results by qRT-PCR. (**a-g**) Total RNAs were isolated from RAW264.7 macrophages infected with H37Rv or BJN followed by autophagy induction by starvation. cDNAs were synthesised and subjected to qRT-PCR analysis as in Figure 2. qRT-PCR results correlated with the RNA-Seq data. Data are means ± SEM from at least three independent experiments; *p < 0.05, **p < 0.01 and ***p < 0.001, all relative to the full control set of 1.0 were determined by one-way ANOVA with Tukey's multiple comparison test.

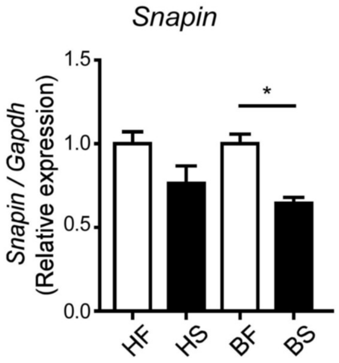

Supplementary Figure S2. *Snapin* expression determined by qRT-PCR. Raw264.7 macrophages were infected with H37Rv or BJN and subjected to autophagy induction by starvation. *Snapin* expression was quantified by qRT-PCR as in Figure 2. Data are means ± SEM from at least three independent experiments; *p < 0.05, relative to the full control set of 1.0 was determined by one-way ANOVA with Tukey's multiple comparison test.

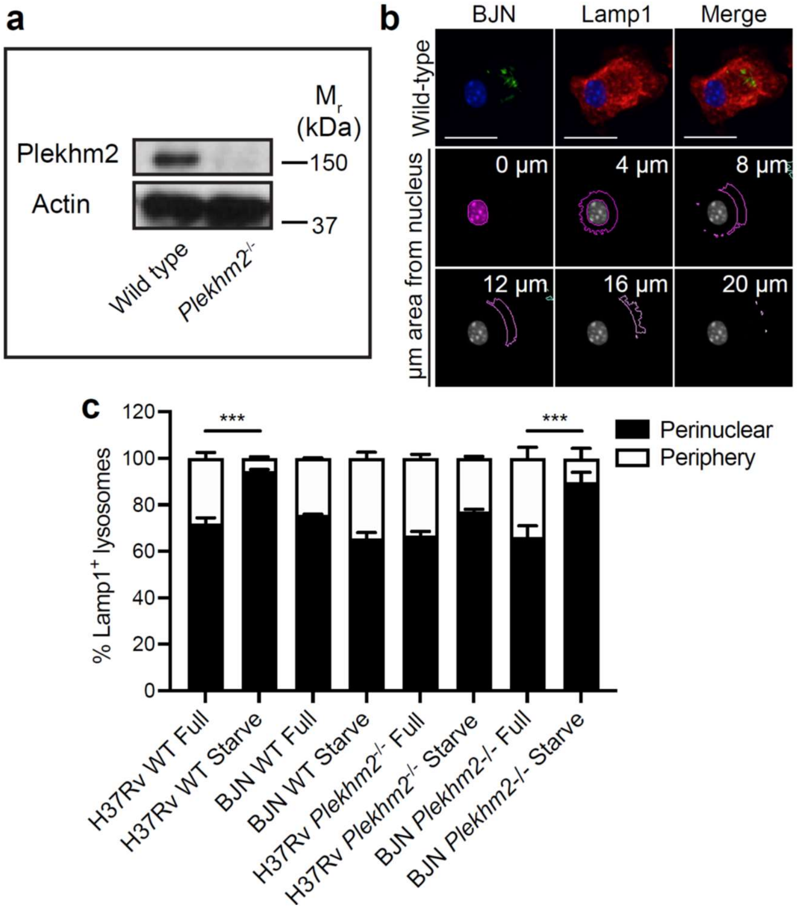

Supplementary Figure S3. Plekhm2 is important for the BJN strain in suppressing lysosome redistribution towards the perinuclear region upon starvation of Hoxb8 macrophages. (**a**) Immunoblot analysis confirmed the lack of Plekhm2 expression in *Plekhm2*$^{-/-}$ knockout Hoxb8 progenitor cells. Representative images cropped from the same blot are shown and full images are included in the supplementary information. (**b-c**) Wild-type and *Plekhm2*$^{-/-}$ progenitors were differentiated into macrophages and subsequently infected with the Alexa-488-labelled *M. tuberculosis* reference strain H37Rv or BJN for 15 min and chased for 1 hr. Host macrophages were then subjected to autophagy induction by starvation for 2 hr. Cells were then fixed and stained for Lamp1, used as a marker for lysosomes. Numbers of lysosomes in different cytoplasmic subareas, as defined by distance from the nucleus in infected cells, were then analysed by high-content image analysis. A representative image from high-content image analysis is shown in (**b**). Magenta lines indicate subareas at different distances from the nucleus. Bar 20 µm. Percent perinuclear Lamp1$^+$ lysosomes (located between 0 and 4 µm from the nucleus) and periphery Lamp1$^+$ lysosomes (located between 4 µm from the nucleus and the cell boundary) were then determined (**c**). Data are means ± SEM from at least three independent experiments; ***$p < 0.001$, relative to the full control was determined by one-way ANOVA with Tukey's multiple comparison test.

Supplementary Tables

Supplementary Table S1. RNA-Seq analysis statistics.

Experiment			Rep	Total reads	% overall read mapping rate	Aligned pairs	Multiple alignments	Discordant alignments	% concordant pair alignment rate
F1	n/a	Full media	1	27,386,449	91.10%	23,745,564	5,413,004 (22.8%)	742,524 (3.1%)	84.00%
F2			2	40,512,990	91.00%	35,429,846	3,867,327 (10.9%)	927,671 (2.6%)	85.20%
S1		Starve media	1	35,874,167	90.80%	31,297,952	7,788,957 (24.9%)	852,917 (2.7%)	84.90%
S2			2	33,734,879	84.40%	27,019,287	3,116,995 (11.5%)	1,156,874 (4.3%)	76.70%
HF1	H37Rv	Full media	1	29,743,686	92.20%	26,383,583	5,582,963 (21.2%)	816,131 (3.1%)	86.00%
HF2			2	30,781,672	91.90%	27,251,546	2,749,183 (10.1%)	847,280 (3.1%)	85.80%
HS1		Starve media	1	31,115,387	91.90%	27,439,608	7,384,534 (26.9%)	919,323 (3.4%)	85.20%
HS2			2	36,904,292	92.20%	32,865,284	3,627,678 (11.0%)	898,018 (2.7%)	86.60%
BF1	BJN	Full media	1	36,539,295	92.70%	32,581,815	6,923,949 (21.3%)	1,102,829 (3.4%)	86.20%
BF2			2	38,958,670	91.10%	34,230,830	3,067,451 (9.0%)	963,351 (2.8%)	85.40%
BS1		Starve media	1	29,004,121	91.30%	25,458,322	7,222,605 (28.4%)	892,075 (3.5%)	84.70%
BS2			2	30,542,595	89.80%	25,754,784	3,247,610 (12.6%)	840,189 (3.3%)	81.60%

Supplementary Table S2. Primer sequences used for qRT-PCR in this study.

Gene name	Primer name	Sequence (5' – 3')
KxDL motif-containing protein 1 (Kxd1)	Kxd1_Fw	5'- TTGGTGGACATGAAACGGGATCT-3'
	Kxd1_Re	5'- CCCTCTGGGATGTGGCTGAAG -3'
Pleckstrin homology domain containing family M (with RUN domain) member 2 (Plekhm2)	Plekhm2_Fw	5'- CAGTCCACGGTTCAGACAGCC-3'
	Plekhm2_Re	5'- CGTGAGGTCTCCATCCTCGC-3'
Syntaxin-3 (Stx3)	Stx3_Fw	5'- TAGTAGTTGTGTTGCTGGGCATT-3'
	Stx3_Re	5'- GCTGGAGTGAAAGCTGGTTATTTC -3'
Autophagy related 4A (Atg4a)	Atg4a_Fw	5'-GGATGTATGCTGCGCTGTGGG-3'
	Atg4a_Re	5'-TCTCCCAGTTCCAATCCCTTCCC-3'
TBC1 domain family member 14 (Tbc1d14)	Tbc1d14_Fw	5'-GACAGAGAGACTTGGGATGGA-3'
	Tbc1d14_Re	5'-GCCTTGTCTTCATATTCCTTCTGG-3'
VPS33B interacting protein apical-basolateral polarity regulator spe-39 homolog (Vipas39)	Vipas39_Fw	5'-TGGAGAACCTGTGGGAAGTATCT-3'
	Vipas39_Re	5'-GGTAGCTGTGTGTAGAAGCTGTT-3'
Phosphoinositide-3-kinase regulatory subunit 5 (Pik3r5)	Pik3r5_Fw	5'-CCATCCTGGCCGATATGTTACTC-3'
	Pik3r5_Re	5'-AAGGTCAGCTCTGTCTGGTAAAC-3'
N-acetylglucosamine-1-phosphotransferase subunits alpha/beta (Gnptab)	Gnptab_Fw	5'-ATGCGGGAAACCCTCGGGAA-3'
	Gnptab_Re	5'-CGGGTCCAGAACAAGCATGGG-3'
NSFL1 (p97) cofactor (p47) (Nsfl1c)	Nsfl1c_Fw	5'-GCAGGAGAAAGGAGGCGGCA-3'
	Nsfl1c_Re	5'-TGGGCATTGGATGGGTCTTGGT-3'
SNAP-associated protein (Snapin)	Snapin_Fw	5'-AGGATCAGAAAGTGGCCCTGGAT-3'
	Snapin_Re	5'-GCCTTAGTCGTTCCTGTGCATTCT-3'
Glyceraldehyde-3-phosphate dehydrogenase (Gapdh)	Gapdh_Fw	5'-GTTGTCTCCTGCGACTTCA-3'
	Gapdh Re	5'-GGTGGTCCAGGGTTTCTTA-3'

Image of immunoblotting

Suppplement Figure S3a

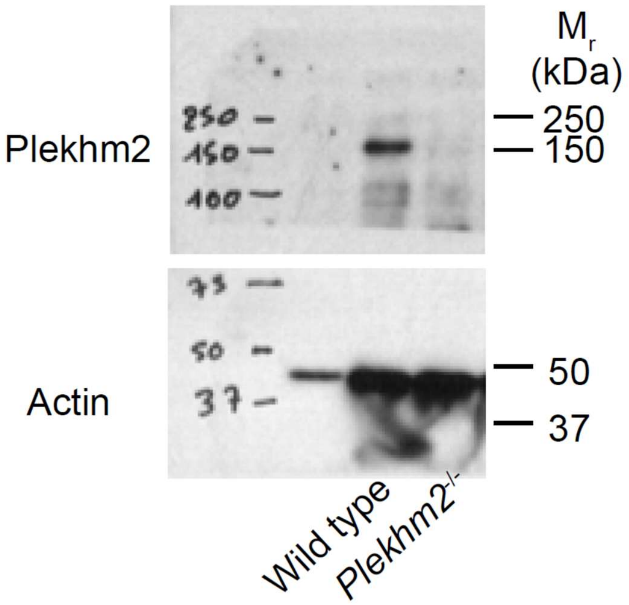

Supplement: Supplementary file 1 — Supplementary Information 1. [file 41598_2021_83835_MOESM1_ESM.pdf]
